# Leaf beetles are ant-nest beetles: the curious life of the juvenile stages of case-bearers (Coleoptera, Chrysomelidae, Cryptocephalinae)

**DOI:** 10.3897/zookeys.547.6098

**Published:** 2015-12-17

**Authors:** Federico A. Agrain, Matthew L. Buffington, Caroline S. Chaboo, Maria L. Chamorro, Matthias Schöller

**Affiliations:** 1 Laboratorio de Entomología, IADIZA, CCT-CONICET, CC507, 5500, Av. A. Ruiz Leal s/n, Pque. Gral. San Martin, Mendoza, Argentina; 2 Systematic Entomology Laboratory, ARS-USDA, MRC 168, National Museum of Natural History, Smithsonian Institution P.O. Box 37012, Washington, DC, 20013-7012, U.S.A.; 3 Division of Entomology, 1501 Crestline Drive, Suite 140, University of Kansas, Lawrence, KS, 66045, U.S.A.; 4 Systematic Entomology Laboratory, ARS-USDA, MRC 168, National Museum of Natural History, Smithsonian Institution P.O. Box 37012, Washington, DC, 20013-7012, U.S.A.; 5 Humboldt-Universität zu Berlin, Faculty of Life Sciences, Lentzeallee 55/57, 14195 Berlin, Germany

**Keywords:** Myrmecophily, Camptosomata, Larvae, Biology, Clytrini, Cryptocephalini

## Abstract

Although some species of Cryptocephalinae (Coleoptera: Chrysomelidae) have been documented with ants (Hymenoptera: Formicidae) for almost 200 years, information on this association is fragmentary. This contribution synthesizes extant literature and analysizes the data for biological patterns. Myrmecophily is more common in the tribe Clytrini than in Cryptocephalini, but not documented for Fulcidacini or the closely-related Lamprosomatinae. Myrmecophilous cryptocephalines (34 species in 14 genera) primarily live among formicine and myrmecines ants as hosts. These two ant lineages are putative sister-groups, with their root-node dated to between 77–90 mya. In the New World tropics, the relatively recent radiation of ants from moist forests to more xeric ecosystems might have propelled the association of cryptocephalines and ant nests. Literature records suggest that the defensive behavioral profile or chemical profile (or both) of these ants has been exploited by cryptocephalines. Another pattern appears to be that specialized natural enemies, especially parasitoid Hymenoptera, exploit cryptocephaline beetles inside the ant nests. With the extant data at hand, based on the minimum age of a fossil larva dated to 45 mya, we can infer that the origin of cryptocephaline myrmecophily could have arisen within the Upper Cretaceous or later. It remains unknown how many times myrmecophily has appeared, or how old is the behavior. This uncertainty is compounded by incongruent hypotheses about the origins of Chrysomelidae and angiosperm-associated lineages of cryptocephalines. Living with ants offers multiple advantages that might have aided the colonization of xeric environments by some cryptocephaline species.

## Introduction

With approximately 40,000 species documented, the Chrysomelidae, commonly called leaf beetles, are one of the most diverse insect groups on Earth. They are well known as phytophages, specializing on all parts of plants, from roots to fruits and flowers. Within this broad lineage, whose origin has been dated from the Middle Jurassic (Wang et al. 2014), numerous instances of plant host-switching have been recorded with clades specialized on certain plant families. In some immature stages, we also find remarkable diet switching to detritivory and myrmecophily (“ant loving”). Such habitat and diet shifts involve behavioral, morphological and chemical changes, yet these are unevenly or poorly studied across Chrysomelidae.

This study focuses on the monophyletic Camptosomata branch of leaf beetles (Gómez-Zurita et al. 2008, and references therein), comprised of two subfamilies: Cryptocephalinae Gyllenhal (127 genera; *ca.* 5300 species) and Lamprosomatinae Lacordaire (13 genera; *ca.* 250 species) (Chamorro 2014a, b). Cryptocephalinae is currently classified into 3 subtribes: Fulcidacini, Clytrini, and Cryptocephalini. Camptosomata has been poorly studied despite its large diversity within Chrysomelidae and several remarkable features in their life cycle, behavior and morphology.

Camptosomates are commonly referred to as “case-bearers” because of the unusual larval behavior of retaining a maternal covering of feces around each egg, carrying and reconstructing it as a protective structure, and ultimately modifying it as a pupation chamber. Schaller (1783) is the first illustrated description of a leaf beetle larvae carrying a case. Since then, Erber’s (1988) synthesis of camptosomate case-bearing behavior has helped us recognize this behavior as a synapomorphy of Cryptocephalinae + Lamprosomatinae. The case functions as a domicile and protective covering from environmental conditions (e.g. desiccation) and/or from predators and parasitoids (e.g., camouflage). The Greek name “camptosomata”, literally “curved body”, was established by Chapuis (1874) (Camptosomes), presumably describing the characteristic J-shape of the soft larva in both Cryptocephalinae and Lamprosomatinae. This curvature results in the anus being located near the legs, so fecal pellets are more easily manipulated in case construction (Erber 1988). Brown and Funk (2005) studied the morphological variation across life stages, construction behavior, and investigated some functions in one species. Two recent treatments on Camptosomata, Chamorro (2014a, b) for adults and a catalog by Chaboo et al. (in press) for juveniles, can now stimulate recognition of new ecological and evolutionary patterns in Camptosomata and aid future research on this major branch of leaf beetles. Chaboo et al. (in press) searched literature records for 354 species (6.7%) of the 5300 recognized camptosomate species, and documented multiple instances of host ant associations (in contrast to the commonly known host plant use of both adult and larvae).

Although myrmecophilous associations can be found in at least 35 beetle families, including varied behavioral and morphological characteristics (Mynhardt 2013), it is not widely known among entomologists, even among ant and leaf beetle biologists, that some Camptosomata appear to seek food and shelter in ant nests. This paper builds on Chamorro (2014a, b) and Chaboo et al. (in press) to further synthesize the literature record, bring focus to the association of some Cryptocephalinae species with ants, and evaluate what pattern, if any, exists.

Chevrolat (1835) was the first to mention case-bearing leaf beetles inside ant nests. Although myrmecophily in leaf beetles has been known for almost 200 years, it remains poorly studied. Within the Chrysomelidae, myrmecophily has evolved in two subfamilies, Eumolpinae, and Cryptocephalinae (Jolivet and Petitpierre 1981, and references therein). Of the 354 species for which some data on juvenile stages exist, only Clytrini and some Cryptocephalini exhibit myrmecophily (Jolivet 1988, Fig. 1 A–D). To date, no case of myrmecophily has been documented for Fulcidacini (the third tribe of Cryptocephalinae) and Lamprosomatinae.

**Figure 1.**
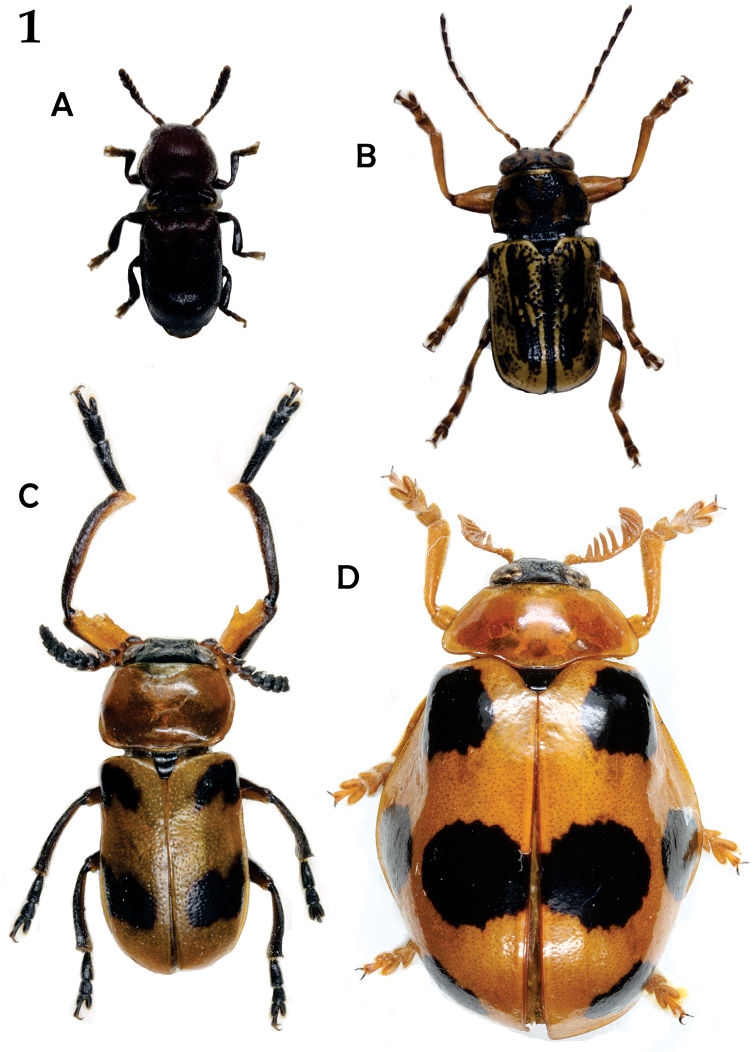
Some Cryptocephalinae associated with ants; **A** *Hockingia curiosa* Selman **B** *Pachybrachis pallidulus* Suffrian **C** *Macrolenes dentipes* (Olivier) **D** *Clytrasoma balyi* Monrós (not to scale). Photos: Matthias Schöller.

Jolivet (1952) published on myrmecophily for *Lachnaia* Chevrolat and *Tituboea* Lacordaire. Brothers et al. (2000) reported that approximately 20 species of Clytrini are commensals in the nests of more than 30 ant species; yet, a small number of these associations are formally documented. Schöller and Witte (2007) hypothesized myrmecophily as a synapomorphy at least for the subtribe Clytrina, however, a phylogeny of this subtribe and the Camptosomata clade and more detailed observations and broader taxon sampling are needed to understand the full picture of these leaf beetle association with ants.

Ants are not the only hosts of some Camptosomata. *Griburius montezuma* (Cryptocephalini) has been reported as living in nets of bird (Beamer 1926) and of packrats *Neotoma* Say & Ord, 1825 (Mammalia: Rodentia; Riley et al. 2001, Riley et al. 2002). *Oomorphus* Curtis (Lamprosomatinae) larvae were also found in packrat nests (Flowers et al. 1994, Riley et al. 2002, Jolivet and Verma 2002). Nothing is known about these peculiar associations; it might be that these larvae were accidentally introduced in these nests (i.e. incorporated through plant materials). However, we cannot ignore that in nature there are several examples of commensals, saprophages, hibernators, occupying these kind of habitats, thus their presence in these nests might indicate a remarkable new association that requires further study. We will not discuss this association further in this paper.

### Challenges of myrmecophily

Ant nests are considered to be well-protected environments, with storage of food items and stable microclimatic conditions. Wilson (1971) and Hölldobler (1972) argued that the insect colony and its immediate environment can be compared to an ecological island, partitioned into many micro-habitats that symbiotic organisms are continuously attempting to colonize. Several myrmecophilous organisms are known to profit from these assets by inhabiting them (Geiselhardt et al. 2007, and references therein). In fact, more than 10,000 social parasite species have been reported in ant nests (Thomas et al. 2005). Coleopterans are common commensals in ant nests (Hölldobler and Wilson 1990). For cryptocephalines, this relationship is based only on the documented presence of the beetle in the ant nest. The degree and quality of the relationship has not been investigated except for a few species for which life histories are known.

The exploitation of ant nests presents some formidable challenges. Wilson (1971) remains the classic overview to non-ants living with ants. Finding the host ants, especially the nest, is the first challenge, but entering the fortress of these aggressive and hostile animals requires behavioral, morphological, and chemical changes to fool the host. A myrmecophile can live outside the nest, being associated with ant trails and even migrating with the ants as they move; they could follow trails or hitchhike on the ants. The host ant can be tricked into carrying invaders inside the nest, and even into feeding them. For example, adult clavigerine staphylinid beetles have glands with oily secretions and associated trichomes that wick the chemicals and fool their host ants into carrying them into the nest and feeding them (Kistner 1982). There may also be great morphological changes to mimic ants, as seen in other beetle groups (e.g. *Myrmex* Sturm (Curculionidae: Otidocephalini). Once accepted inside the ant nest, the ‘uninvited guest’ insect has some trophic options as predators (of ants or other arthropod inhabitants), scavengers (nest refuse, dead bodies), fungivores, herbivores (eating stored grain and leaves), parasitoids, and thieves (e.g. regurgitated food). An invading insect can also choose from a variety of places or niches to live, such as refuse piles, storage chambers, and brood chambers. Research is more advanced on other insect mymecophiles (e.g. Carabidae: Paussinae; Staphylinidae) and may provide useful models to guide research on the cryptocephaline myrmecophiles.

## Materials and methods

Existing literature on ant-camptosome associations was synthesized (Table 1). Beetle names were validated in Chaboo et al. (in press), with family- and genus-group names and authors following Bouchard et al. (2011), Löbl and Smetana (2010), and Seeno and Wilcox (1982). Ant names were validated using AntWeb (http://www.antweb.org). In order to trace the evolution of cryptocephaline myrmecophiles and their hosts, existing information on the phylogenetic patterns among Formicidae was extracted from Moreau and Bell (2013), Brady et al. (2006), Rabelling et al. (2008), and Schultz and Brady (2008). Author’s names for all genera and species of ants and beetles cited in the text are given in Table 1.

**Table 1.** Known ant host of Cryptocephalinae. CL=Clytrini, CR=Cryptocephalini.

Taxon	Beetle species	Tribe	Source
**Dolichoderinae ants**			
*Tapinoma erraticum* Latreille	*Labidostomis taxicornis* (Fabricius)	CL	Barbier 1976, Erber 1988
**Dorylinae ants**			
*Dorylus* sp.	Clytrinae larvae follow the migrations of their hosts outside the nest during day or night	CL	Jolivet 1952
**Formicinae ants**			
*Camponotus* sp.	Clytra (Clytra) laeviuscula Ratzeburg	CL	Paulian and Villiers 1939
*Camponotus ligniperdus* Latreille	Clytra (Clytra) quadripunctata (L.)	CL	Wasmann 1894a, Skwarra 1927, Jolivet 1952, Medvedev 1962
Camponotus (Latreille)	*Clytra* sp.	CL	Medvedev 1962, Lee and Morimoto 1991, Jolivet and Hawkeswood 1995
*Camponotus melleus* Say	*Coscinoptera dominicana dominicana* (Fabricius)	CL	Wasmann 1894a, Cockerell (1891)
Camponotus (Myrmosericus) rufoglaucus Jerdon	*Hockingia curiosa* Selman	CL	Selman 1962
*Camponotus* sp.	*Clytrasoma maschwitzi* Schöller	CL	Schöller and Witte 2007
*Camponotus* sp.	Clytra (Clytra) quadripunctata (L.)		
*Camponotus*	Clytrine	CL	Jolivet 1978
*Cataglyphis cursor* Fonscolombe	Clytra (Clytraria) atraphaxidis (Pallas)	CL	Xambeu 1899, Jolivet 1952
*Cataglyphis bicolor* (Fabricius)	Clytra (Clytraria) atraphaxidis (Pallas)	CL	Medvedev 1962
*Cataglyphis cursor* Fonscolombe	Clytra (Clytraria) atraphaxidis (Pallas)	CL	Jolivet (1952)
*Cataglyphis bicolor* Fabricius	Clytra (Clytraria) atraphaxidis (Pallas)	CL	Xambeu (1899), Medvedev (1962)
*Cataglyphis* Förster	*Clytra* sp.	CL	Medvedev 1962, Lee and Morimoto 1991, Jolivet and Hawkeswood 1995
*Cataglyphis cursor* Fonscolombe	Lachnaia (Lachnaia) tristigma (Lacordaire)	CL	Medvedev 1962
*Cataglyphis cursor* Fonscolombe	Lachnaia (Lachnaia) tristigma (Lacordaire)	CL	Xambeu 1899
*Cataglyphis* Förster	*Lachnaia* Chevrolat in Dejean	CL	Jolivet and Hawkeswood 1995
*Formica pallidefulva* Latreille	*Anomoea flavokansiensis* Moldenke	CL	Stiefel et al. 1995, LeSage and Stiefel 1996, Stiefel and Margolies 1998
*Formica sanguinea* Latreille	Clytra (Clytra) laeviuscula Ratzeburg	CL	Wasmann 1894a, Donisthorpe 1927, Jolivet 1952, Lapeva-Gjonova 2013
*Formica pratensis* DeGeer	Clytra (Clytra) laeviuscula Ratzeburg	CL	Wasmann 1894a, Skwarra 1927, Jolivet 1952
*Formica fusca* L.	Clytra (Clytra) laeviuscula Ratzeburg	CL	Reineck 1928, Erber 1988
*Formica rufa* L.	Clytra (Clytra) quadripunctata (L.)	CL	Chevrolat 1835, Märkel 1841, Rosenhauer 1842, Brauns 1864, Collet 1883, Fowler 1890, Wasmann 1894a, Donisthorpe 1908, Donisthorpe 1927, Skwarra 1927, Jolivet 1952, Medvedev 1962, Lapeva-Gjonova 2013
*Formica rotundata* Klug	Clytra (Clytra) quadripunctata (L.)	CL	Medvedev 1962, Erber 1988
*Formica exsecta* Nylander	Clytra (Clytra) quadripunctata (L.)	CL	Brauns 1864, Wasmann 1894b, Skwarra 1927, Jolivet 1952, Medvedev 1962
Possibly *Formica congerens* Nylander	Clytra (Clytra) quadripunctata (L.)	CL	Hislop 1872
*Formica sanguinea* Latreille	Clytra (Clytra) quadripunctata (L.)	CL	Wasmann 1894a, Skwarra 1927, Jolivet 1952, Medvedev 1962, Medvedev 1962
*Formica pratensis* DeGeer	Clytra (Clytra) quadripunctata (L.)	CL	Skwarra 1927, Jolivet 1952, Wasmann 1894a, Erber 1988
*Formica rufo-pratensis* Forel	Clytra (Clytra) quadripunctata (L.)	CL	Skwarra 1927, Erber 1988
*Formica pressilabris* Nylander	Clytra (Clytra) quadripunctata (L.)	CL	Skwarra 1927, Erber 1988
*Formica gagates* Nylander	Clytra (Clytra) quadripunctata (L.)	CL	Wasmann 1894a, Skwarra 1927, Jolivet 1952, Medvedev 1962
*Formica uralensis* Ruzsky	Clytra (Clytra) quadripunctata (L.)	CL	Skwarra 1927, Medvedev 1962
*Formica* sp.	Clytra (Clytra) quadripunctata (L.)	CL	Priefert 1926, Selman 1988
*Formica rufa* L.	*Clytra* sp.	CL	Chevrolat 1835
*Formica* L.	*Clytra* sp.	CL	Medvedev 1962, Lee and Morimoto 1991a
*Formica fusca* L.	*Clytra* sp.	CL	Medvedev 1962
*Formica fusca* L.	*Clytra* sp.	CL	Samšiṅák 1956, Medvedev 1962
*Formica* sp.	*Coscinoptera dominicana dominicana* (Fabricius)	CL	Riley 1874b
*Formica obscuripes* Forel	*Coscinoptera dominicana dominicana* (Fabricius)	CL	Riley 1874b
*Formica selysii* Bondroit	*Pachybrachis anoguttatus* Suffrian (found inside the ant nest)	CR	Schöller 1995
*Formica fusca subaenescens* Emerton	*Coscinoptera dominicana dominicana* (Fabricius)	CL	Jolivet 1952, Wasmann 1894a
*Formica obscuripes* Forel	*Coscinoptera dominicana dominicana* (Fabricius)	CL	Cockerell 1891, Jolivet 1952, Riley 1874
*Formica neoclara* Emery	*Coscinoptera dominicana franciscana* (LeConte)	CL	Slosser 2003
*Formica fusca subaenescens* Emerton	*Coscinoptera vittigera* (LeConte)	CL	Wasmann 1894a, Jolivet 1952
*Formica fusca* L.	*Coscinoptera vittigera* Probably *Coscinoptera dominicana* (Fabricius)	CL	Cockerell 1891, Wasmann 1894a, Jolivet 1952
*Formica*	Clytrine	CL	Jolivet 1978
*Lasius niger* L.	Cryptocephalus (Burlinius) ocellatus ocellatus Drapiez	CR	Schöller 1995
*Lasius niger* L.	Clytra (Clytra) laeviuscula Ratzeburg	CL	Fiori 1948, Wasmann 1894a, Erber 1988
*Lasius alienonigra* Forst.	Clytra (Clytra) laeviuscula Ratzeburg	CL	Skwarra 1927, Erber 1988
*Lasius alienus* Forst.	Clytra (Clytra) laeviuscula Ratzeburg	CL	Skwarra 1927, Fiori 1948, Erber 1988
*Lasius neglectus* Van Loon, Boomsma & András-Falvy	Clytra (Clytra) laeviuscula Ratzeburg	CL	Selman 1988, Espadaler and Bernal 2009
*Lasius niger* L.	Clytra (Clytra) laeviuscula Ratzeburg	CL	Donisthorpe 1927
*Lasius niger* L.	Clytra (Clytra) laeviuscula Ratzeburg	CL	Donisthorpe 1927
*Lasius flavus* (DeGeer)	Clytra (Clytra) quadripunctata (L.)	CL	Wasmann 1894a, Skwarra 1927, Jolivet 1952, Medvedev 1962
*Lasius* Latreille	*Clytra* sp.	CL	Medvedev 1962, Lee and Morimoto 1991, Jolivet and Hawkeswood 1995
*Lasius neglectus* Van Loon, Boomsma & András-Falvy	Clytrinae larvae	CL	Nagy et al. 2009
*Lasius fuliginosus* Latreille	Cryptocephalus (Burlinius) fulvus fulvus (Goeze)	CR	Donisthorpe 1927
*Plagiolepis* sp.	*Tituboea macropus* (Illiger)	CL	Medvedev 1962, Erber 1988
**Myrmicinae ants**			
*Aphaenogaster subterranea* Latreille	Clytra (Clytra) laeviuscula Ratzeburg	CL	Fiori 1948, Erber 1988
Aphaenogaster (Myrmica) testaceopilosa Lucas	*Tituboea octosignata* (Fabricius)	CL	Lucas 1850
*Aphaenogaster testaceopilosa* Lucas	Cryptocephaline	Undetermined	Wasmann 1894a, Xambeu 1899, Medvedev 1962, Jolivet 1952
*Atta mexicana* (F. Smith) (Larvae saprophagous)	*Megalostomis dimidiata* Lacordaire	CL	Rojas 1989, Navarrete-Heredia (2001) (as accidental)
*Atta* Fabricius	*Megalostomis dimidiata* Lacordaire	CL	Moldenke 1970
*Atta* nest (*digging on*)	*Megalostomis dimidiata* Lacordaire	CL	Agrain 2010
*Atta texana* (Buckley)	*Megalostomis dimidiata* Lacordaire (as *Megalostomis major* Crotch). 2.5m depth.	CL	Waller and Moser 1990, Della Lucia 1993, Navarrete-Heredia 2001 (as accidental)
*Atta*	Clytrine	CL	Jolivet 1978
*Atta mexicana* (F. Smith)	*Pachybrachis* sp. On external ant debris	CR	Márquez-Luna and Navarrete-Heredia 1994, Navarrete-Heredia 2001 (as accidental)
*Atta mexicana* (F. Smith)	*Griburius* sp. (misspelled as *Griburium*). On external ant debris,	CR	Márquez-Luna and Navarrete-Heredia 1994, Navarrete-Heredia 2001(as accidental).
*Crematogaster lineolata* (Say)	*Anomoea*	CL	Stiefel and Margolies 1998
*Crematogaster lineolata* Say (the ants carries the eggs to their nest)	*Anomoea flavokansiensis* Moldenke	CL	Stiefel and Margolies (1998)
*Crematogaster mimosa* Santschi	*Hockingia curiosa* Selman	CL	Selman 1988, Erber 1988, Jolivet 1991
*Crematogaster sjostedti* Mayr	*Hockingia* Selman	CL	Selman 1962
Crematogaster (Crematogaster) nigriceps Emery	*Isnus petasus* Selman	CR	Selman 1962, 1988; Hocking 1970, Jolivet 1986
Nest of *Crematogaster peringueyi* Emery	Clytrine cases	CL	Péringuey (1898)
*Crematogaster* sp.	*Coenobius macarangae* Gressitt (living on myrmecophyte)	CR	Jolivet 1991
*Crematogaster* sp.	*Cadmus macarangae* Gressitt (living on myrmecophyte)	CR	Jolivet 1991
*Messor clivorum sevani* Kar.	Clytra (Clytraria) valeriana valeriana Ménétriés	CL	Medvedev 1962
*Messor* Forel	*Clytra* sp.	CL	Jolivet and Hawkeswood 1995
*Messor barbarus* L.	*Lachnaia vicina* Lacordaire.	CL	Barbier 1976, Erber 1988
*Messor barbarus capitatus* Latreille	*Tituboea biguttata* (Olivier)	CL	Fiori 1957
*Messor* spp.	*Tituboea biguttata* (Olivier)	CL	Erber 1988
*Messor barbarus* L.	*Tituboea biguttata* (Olivier)	CL	Medvedev 1962, Jolivet 1952
*Messor barbarus capitatus* Latreille	*Tituboea biguttata* (Olivier)	CL	Medvedev 1962
*Messor barbara* (L.)	*Tituboea* Lacordaire	CL	Barbier 1976
*Messor barbara* L.	Clytrine	CL	Barbier 1976
*Mymica rugolosa* Nylander, queen using larval case for colony founding	*Cryptocephalus morarei* (L.)	CR	Schöller 1999
*Pheidole* sp. Queen with eggs and workers on larval case	*Cryptocephalus anceps* Suffrian	CR	Schöller 1999
*Tetramorium caespitum* L.	*Clytra* sp.	CL	Girard 1873
*Tetramorium caespitum* L.	*Clytra* sp.	CL	Escherich and Emery 1897, Jolivet 1952, Medvedev 1962
*Tetramorium caespitum* L.	*Smaragdina concolor* (Fabricius)	CL	Xambeu 1899, Medvedev 1962, Erber 1988
*Tetramorium vespitum* L.	*Smaragdina concolor* (Fabricius)	CL	Jolivet 1952
*Leaf cutting ant nest*	*Megalostomis dimidiata* Lacordaire	CL	Agrain 2010
**Undetermined ants**			
Eat detritus and Humus, associated with ants	*Anomoea*	CL	Moldenke 1970, Jolivet and Hawkeswood 1995
Myrmecophile	*Clytra* sp.	CL	Escherich 1899, Erber 1988
Ant eggs	Clytrine	CL	McAtee 1932
As myrmecophiles	Clytrine	CL	Jolivet 1952
As myrmecophiles	Clytrine	CL	Doguet 1992
Dead leaves in ant nests	Clytrine	CL	Johnson and Triplehorn 2004
In ant nests	Clytrine	CL	Jolivet 1991
Larvae that overwinter as ant inquilines	Clytrine	CL	Erber 1988, Steifel et al. 1995, Jolivet 1996
Ant host	*Coscinoptera dominicana dominicana* (Fabricius)	CL	Riley 1882, Wasmann 1894a, Van Dyke 1925, Wickham 1896
Ants on *Acacia* tolerate ants	Cryptocephaline	CR	Jolivet 1996
Ant nests	*Cryptocephalus* Geoffroy	CR	Donisthorpe 1927, Kasap and Crowson 1976
Ant host	*Helioscopa* Gistel	CL	Jolivet and Hawkeswood 1995
Associated with ants, myrmecophiles, or submyrmecophiles	*Labidostomis* Chevrolat in Dejean	CL	Jolivet 1952, 1978; Selman 1988, Erber 1988
As obligate or facultative ant inquilines	Lachnaia Chevrolat in Dejean	CL	Erber 1988
Myrmecophilous larvae	*Lachnaia italica italica* Weise	CL	Regalin et al. 2006
Found in the vicinity of ant nest)	*Macrolenes dentipes* Olivier	CL	Schöller 1998
As myrmecophiles	*Megalostomis* Chevrolat	CL	Jolivet and Hawkeswood 1995
Found in the vicinity of ant nest)	*Pachybrachis anoguttatus* Suffrian	CR	Schöller 1998
In ant nests	Saxinis (Boreosaxinis) saucia LeConte	CL	Van Dyke 1925, Hatch 1971

Terminology: we use the terms ‘myrmecophily’ and ‘myrmecophilous’ in a broad sense, meaning casual or intimate association of the beetle with ants. Such interactions could involve different forms, from mutualism (benefits for both associates), parasitism (host resources necessarily drained for parasite’s own reproduction), commensalism (with no direct effect on the host), predation (direct feeding/damage to the host), or inquilinism (the ‘guest’ obtains shelter and other resources from the host without damaging host). For extended definitions and discussion of the latter terms see Parmentier and Michel (2013 and references therein). Myrmecophiles are often differentiated as ‘protective’ or ‘symphiles’ according to their strategies (Geiselhard et al. 2007 and references therein). We also follow Mynhardt (2013 and references therein), as a glossary and modern critique of the terms historically used in the study of myrmecophily.

Figure 1 photographs were taken by Matthias Schöller with a Nikon D5100 mounted on a stereo microscope, and the photos stacked with CombineZP software (http://www.hadleyweb.pwp.blueyonder.co.uk).

## Results

Our synthesis of the literature reveals that 34 species of Cryptocephalinae have been associated with ants. The following ant groups host cryptocephalines: Dolichoderinae (1 species), Dorylinae (1 species) Formicinae (13 species), Myrmicinae (16 species); 11 species were reported on an undetermined host ant. Of the latter, 4 cryptocephaline genera are included in this category for which there is no other ant record. These associations represent approximately 0.6% of current species diversity of Cryptocephalinae. In summary, 14% of the 127 Cryptocephalinae genera are associated with ants at some level, as truly myrmecophilous. We found several previously unnoticed patterns in the ant associations of camptosomate genera and species. Regarding the extent of myrmecophily within Camptosomata, we found that ant associations are documented only in two tribes, Clytrini and Cryptocephalini (Fig. 1). Both tribes show distinct patterns of ant association — clytrines appear to be able to exploit several subfamilies of ants, including Formicinae, Myrmicinae, Dolichoderinae, and Dorylinae, while cryptocephalines appear to be restricted to the Formicinae and Myrmicinae.

**Figure 2.**
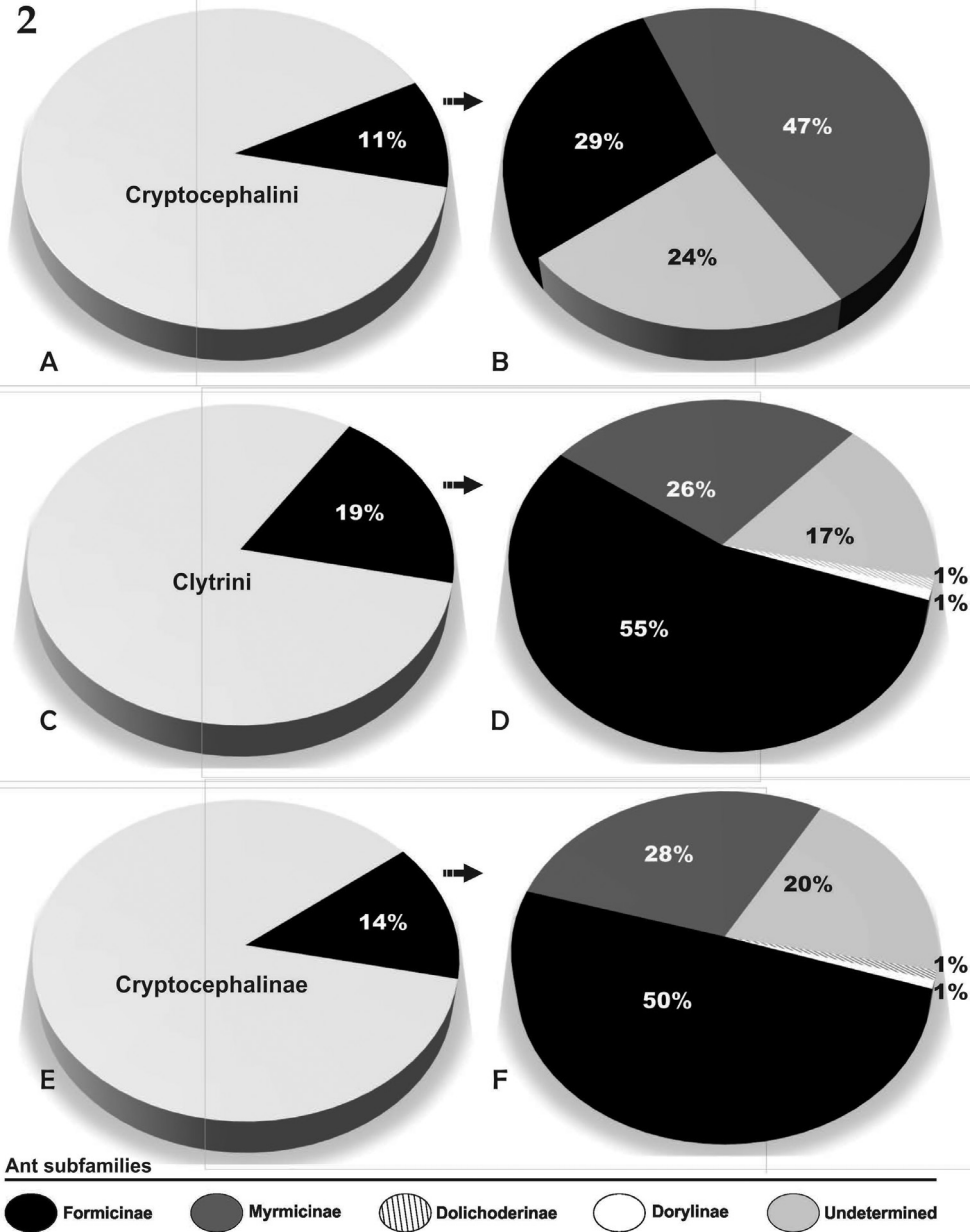
Ant association records. Each pie chart indicates ant association records expressed as percentages, different color tones refer to each ant subfamilies as indicated in the color reference below: **A–B** records within the tribe Cryptocephalini **C–D** records within the tribe Clytrini **E–F** records within the whole subfamily.

Data extracted from literature and synthesized here suggest that myrmecophily in Cryptocephalinae is rare or simply unknown, being more frequent in Clytrini (Fig. 2 A–F). Cryptocephalini is currently composed of 54 genera (Chamorro 2014b). Out of these, six genera of Cryptocephalini (11%), have records of association with ants, while Clytrini has 62 genera (Chamorro 2014b), with 12 genera (19%) associated with ants (Table 2). Within the Clytrini, almost all documented cases of myrmecophily are in the subtribe Clytrina, except for two genera in the subtribe Megalostomina (i.e. *Coscinoptera*, and *Megalostomis*) and two genera in Babiina (*Helioscopa*, *Saxinis*).

**Table 2.** The number of Cryptocephalinae genera associated with ant hosts.

Tribe / Genus	Myrmecophilous species number	Number of ant genera recorded as host
**Clytrini**		
*Anomoea* Agassiz	1 + undet.	2 + undet.
*Clytra* Laicharting	4 + undet.	7 + undet.
*Clytrasoma* Jacoby	1	1
*Coscinoptera* Lacordaire	2	2 + undet.
*Helioscopa* Gistel	undet.	undet.
*Hockingia* Selman	1 + undet.	2
*Labidostomis* Germar	1 + undet.	1 + undet.
*Lachnaia* Chevrolat	1 + undet.	2 + undet.
*Macrolenes* Chevrolat	1	undet.
*Megalostomis* Chevrolat	2 + undet.	2 + undet.
*Saxinis* Lacordaire	1	undet.
*Smaragdina* Chevrolat	1	1
*Tituboea* Lacordaire	3 + undet.	3
Undetermined	11	5 + 6 undet.
**Cryptocephalini**		
*Cryptocephalus* Geoffroy	1 + undet.	1 + undet.
*Cadmus* Erichson	1	1
*Coenobius* Suffrian	1	1
*Griburius* Haldeman	1 undet.	1
*Isnus* Weise	1	1
*Pachybrachis* Chevrolat	1 + 2 undet.	2 + undet.
Undetermined	2	1 + undet.

## Discussion

Since most records found in the extant literature are the product of a chance finding of the beetles in association with the ant nests, and not of a directed search, it not clear how widespread ant associations really are. Below we discuss some patterns of ant associations we recognized in our synthesis. The study of myrmecophilous beetles have has revaled an extraordinary amount of adaptations (Mynhardt 2013), therefore, further research on other beetle lineages may provide useful touchstones to guide theoretical or empirical research on cryptocephaline myrmecophily.

*Quality of available data.* Records of myrmecophilous species summarized in Table 1 have accumulated over the last 180 years since Chevrolat (1835) first reported a cryptocephaline-ant association. We believe a collection bias exists in these data. For instance, some ant nests are the focus of a great deal of study and subsequent excavation (e.g. *Atta* nests); these species would, as a result, have more records of myrmecophilous species inhabiting them than other ant nests that are more difficult to study (e.g. *Dorylus*) or simply understudied. Verifying the accuracy of host records presented here has not been our primary goal, so some records presented here may not hold up to future scrutiny and/or data (through additional fieldwork and/or examination of museum vouchers). Finally, some species of cryptocephalines have received more study than others, and hence, their host patterns are not in common with other species. For instance, the Palearctic Clytra (Clytra) quadripunctata is one of the most studied cryptocephaline species, and has consequently been recorded from ant nests of 12 species within 3 genera in 2 different subfamilies. If more fieldwork were conducted on all myrmecophilous cryptocephaline species, the pattern of host use might be very different.

*Taxonomic patterns of host use.* All records examined here indicate that myrmecophilous cryptocephalines are specialists on the formicoid ants, a branch of Formicidae, which include the most common ant species as well as the major invasive species (Brady et al. 2006). The sister-group to the formicoids, the poneroid clade, does not appear to be exploited by cryptocephalines. This formicoid clade has been estimated to range between 105–120 mya (Brady et al. 2006) and 107–119 (Moreau and Bell 2013), but the subfamilies and genera of interest to this study are much younger, and will be covered below.

The most striking pattern that emerges from the assembled data relates to *Clytra*. Members of this genus have the broadest host range, with records from some of the more core formicines such as the carpenter ants (*Camponotus*) and *Formica* ants, but also from the myrmicines *Aphaenogaster*, *Messor*, and *Tetramorium*. While many species of *Camponotus* and *Formica* can be found commonly in forested environments (and often, in fact, co-occur), species of *Messor* are found in more xeric environments, and are herbivorous, seed-harvesting ants (the former are generalist predators). However, Brady et al. (2006) estimated the root-nodes of formicines and myrmicines to be roughly 77–82 mya, and concluded these two subfamilies are sister-groups of each other. Moreau and Bell (2013) recovered the root node of formicines at between 74.9 and 90.3 mya, and myrmecines between 79.3 and 89.8 mya; this study did not recover the two subfamilies as sister-groups, but instead found ectatommines and heteroponerines nested between the two families (but lacking branch support). Today cryptocephalines are associated with both the formicines and myrmecines. Within *Clytra*, we see some degree of host specificity, exemplified by Clytra (Clytra) laeviuscula having an affinity for species of *Lasius*, Clytra (Clytra) quadripunctata being associated with species of *Formica*, and Clytra (Clytraria) atraphaxidis being recovered only from species of *Cataglyphis* (Table 1). This last observation suggests that something of the ant’s defensive behavioral profile or chemical profile (or both) have been exploited by these beetles, and through this exploitation, a certain degree of evolutionary canalization has taken place. Otherwise, we would likely see a plethora of generalist beetles exploiting all manners of ant subfamilies; the data herein reported do not support that hypothesis.

Below we summarize some of the broad challenges of myrmecophily mentioned above (see (Hölldobler and Wilson 1990). Myrmecophilous cryptocephalines appear to use different strategies to live near, enter, or inhabit ant nests. The following discussion analyzes the quality of data available for each challenge posed by the ants’ well-defended fortress.

1) *Finding the host ant.* In some Clytrini, the female oviposits on a leaf and drops the egg, after being intricately covered by feces, to the ground. Ants then carry the eggs, or the first instar larvae within its fecal case, inside the nest (Jolivet 1991, Schöller 2011). Some egg cases are attached to the substrate by a peduncle; Jolivet and Petitpierre (1981) mentioned that ants cut the peduncle in those cases. They also remark that ‘naked’ eggs, i.e. lacking a case, are eaten by the ants.

2) *Living outside the nest.* Some species have been found on external ant debris and are known to feed on it. The biologic meaning of this have not yet been studied, it might be that debris is a rich food source, or possibly, it provides the beetle or larvae with some sort of camouflage.

3) *Trail following.* Jolivet (1952) mentioned that Clytrini larvae follow the migrations of the nomadic *Dorylus* ants (army ants, Formicidae: Dorylinae) during day or night, and that the clytrine adults interact with ants near or inside the nest. This suggests that in some cases, all life stages of camptosomates are capable of interacting with ants. In the case here, the presence in the adults of deep grooves for the reception of appendages (such as antennae and legs) and the cryptic nature of the presence of their head within the prothorax, suggests this to be true.

4) *Entering the ant nest.* All myrmecophiles must enter and remain in the ant nests without being expelled or killed (Hölldobler and Wilson 1990, Lenoir et al. 2001). The inquiline must be adopted, or at least tolerated inside the nest, and this is usually accomplished by chemical mimicry [e.g. *Trichopsenius* Horn (Coleoptera: Staphylinidae) inside *Reticulitermes* Holmgren nests (Isoptera: Rhinotermitidae) (Lenoir et al. 2001, and references therein)]. Erber (1988) indicates that *Hockingia curiosa* might live obligatorily in ants nest, the genus mimics the body form of *Crematogaster* ants in Tanzania. The adults exhibit interesting myrmecophilous organs, which consist of two setae bearing tubercles on the pronotum (Fig. 1A), possibly with a secretory function. *Isnus petasus* also bears long setae on the lateral pronotal margins (Selman 1962). Secretory hairs or bristles and special glands (e.g. adoption glands *sensu* Hölldobler 1970 or defense glands) are not uncommon in myrmecophilous insects (Hölldobler and Wilson 1990) and they may discharge mimicking, appeasing, tranquilizing, repellent or deadly chemicals. Detailed anatomical and scanning electron microscopy might reveal such organs in Cryptocephalinae and chemical analysis will reveal the nature of the exudates. Cryptocephaline adults studied thus far bear a paired basolateral seta on the pronotum.

5) *Evolution towards living and surviving in ant nests.* Once inside an ant nest, whether temporarily or long-term, every myrmecophile is faced with new challenges, from avoiding being detected as an enemy, to finding a safe micro-habitat within the ant complex, to finding food, and carrying on its life cycle.

5a) *Avoiding being eaten by ants.* Cryptocephalinae adults exhibit some typical chrysomelid defenses – chemical sequestration and secretion of toxic compounds (e.g. reflex bleeding) (Selman 1988), mimicry (Hespenheide 1999), defensive stridulation (Monrós 1953, Schmitt 1994), thanatosis (Monrós 1953, Selman 1988), aposematic coloration (Selman 1988, and references therein), gregarious behavior (Monrós 1953); and, as mentioned above, at least some species are thought to have complex myrmecophilous organs (Selman 1988). Egg, larval and pupal cases provide physical protection against predators (Wallace 1970, Root and Messina 1983, Schöller 2014). Furthermore, they are also cryptic and work as a barrier against desiccation, this last function being important as it has been observed that camptosomates are relatively more sensitive to desiccation than other chrysomelids (Root and Messina 1983). The larval cases are sometimes armored with plant trichomes (Chaboo et al. 2008). Camptosomata that develop within the ant nest have to leave it as adults, and are consequently faced with attacks by ants. Species that develop in the vicinity of the nest may be attacked as well. Clytra (Clytra) laeviuscula responds with thanathosis when attacked by ants and when the ants lose interest in the beetle, it walks away or flies off quickly. The adults of *Labidostomis taxicornis* and *Lachnaea pubescens* do not exhibit thanathosis; instead, they move quickly to avoid being attacked, and typically do not survive the ant attacks if caught (Schöller pers. obs.). Nevertheless, defense or antipredator devices of the larvae against ant attacks largely remain unknown.

5b) *Avoiding other dangerous organisms in the ant nest.* In reports of associations with ants, hymenopterans are the most frequent parasitoids of eggs and larvae of the Camptosomata (Erber 1988). Bethylidae wasps are known specialized parasitoids of coleopteran larvae (Evans 1964); many bethylid females are wingless, and their small size and flat body allow them to locate prey in hard-to-access places, such as ant nests. Nagy (1969) found the bethylid *Mesitius horvathi* Kieffer attacking a clytrine. Mutillids (velvet ants; Mutillidae) were also found to attack the enclosed larvae or pupae of species of Coleoptera, including some cryptocephalines (Lawrence and Britton 1991) that live in the ant nests (Brothers et al. 2000). Brothers (1975) could not trace a parallel between the evolution of the mutillids and that of their hosts, so colonization of new hosts is likely a common pattern. Mutillids are thought to have evolved from parasitoids of ground-nesting Hymenoptera, however, host fidelity is not high and host-shifts to distantly related hosts (even in different orders) have apparently occurred. Brothers et al. (2000) indicate that more host records of mutillids parasitizing Clytrini are expected to be found, while the occurrence of Cryptocephalini species as hosts of mutillids in association with ants need to be confirmed. The larvae of mutillid wasps may be parasitoids of host stages which are enclosed in some sort of “hard” package and which are not actively feeding (Brothers 1972, 1989). In ant associations, the well-known occurrences of mutillids always involve parasitism of chrysomelid beetles living in the ant nests and having hard cases. It is unknown whether other mutillid species attack ants directly.

Within the proctotrupomorph Hymenoptera (most of the formerly recognized superfamilies of Parasitica; Sharkey et al. 2007) are a number of ant-specialist lineages. The Universal Chalcidoidea Database (Noyes 2015) cites just under 700 parasitic chalcidoid species that have been recorded from Chrysomelidae; among these species, five have been recorded from *Cryptocephalus*, and none from the other myrmecophilous genera covered herein. Within Ichneumonoidea, 77 genera and over 200 species are known parasitoids of chrysomelids, with 7 species of *Cryptocephalus* recorded as hosts (Yu et al. 2012), including *Gelis acarorum* (L.) and *Dimophora evanialis* Gravenhorst (Schöller 1999). Together with members of the aculeate Bethylidae and Mutillidae, the parasitoid pressure on cryptocephalines to escape parasitism is rather high, and utilizing a refugia such as an ant nest would release the parasitoid pressure significantly on these beetles.

5c) *Microhabitat specialization within the nest and diet*: An ant nest presents multiple places to live, including open chambers, refuse heaps (“kitchen midden”), brood chambers or nurseries (heavily defended but high-quality food), and fungus gardens for those ants that cultivate fungi. Presently it is unknown where cryptocephaline myrmecophiles live within the ant nest. Some of these sites can offer different degrees of protection and different resources to exploit, yet nothing is known about the selection mechanism employed by myrmecophilous cryptocephalines among the different nest chambers. The only insight may be provided by the relation between the food inside the ant nest and the diet of the beetle larvae. Leaf beetles show a general pattern of adults and larvae living on the same host plants. However, some cryptocephalines show a further distinction where the larval and adult stages can have different habitats and diet; this is particularly true for Clytrini and Cryptocephalini. Some species have zoosaprophagous and phytosaprophagous larvae (Schöller 1998). The myrmecophilous larvae appear to feed on ant droppings and pellets (Erber 1988; Lapeva-Gjonova 2013); others feed on detritus (LeSage 1985, 1986; Erber 1988, Stiefel 1993), leaf litter or dead insects collected by the ants (Schöller 1998); or, on ants or ant bodies (Riley 1882). Schöller (1995) posits that zoosaprophagy may be an important aid for the development of larvae inhabiting arid and semi-arid regions, when plant foliage is scarce. Even if some interactions between ants and their associates are known (i.e. social parasitism), the precise behavioral characters and payoffs are not so clear. It is believed that most of the nest associates take advantage of the nest micro-habitat, living as commensals on dead ants and assorted detritus from the ant fungus garden (Waller and Moser 1990).

Fungi inside an ant nest can provide food or can pose a threat to cryptocephalines. Ants such as the Attini (the leafcutter ants) cultivate fungi and these fungus gardens may provide both a micro-habitat to live in and a larder of food. Fungi are commonly known to negatively affect immature stages of cryptocephalines. Yet, there is only one formal citation by Jolivet and Theodorides (1952) of the fungus *Sporotrichum* sp. affecting the larvae and pupae of Clytra (Clytra) quadripunctata; currently it is impossible to know the specificity of this relationship. Fungal mycelia appear to be part of the case construction (Chaboo et al. 2008).

6.) *Benefits for the host*? Although no chemical recompense is known to be offered by myrmecophilous cryptocephalines, Schöller (1999) found empty larval cases of *Cryptocephalus* species being used as microchorions by certain Formicidae and Aranae. Yet more remarkable, Schöller (1999) also found queens of *Myrmica* and *Pheidole* using cases as a place for colony founding, which indicates that these myrmecophilous systems are rather complex and may be synergistic. However, it should be noted that most strategies of cryptocephalines to enter, reside and leave the ant nests remain largely undocumented.

*Strength of host association.* The strength of myrmecophilic relationships can vary, as some larvae can survive without actually entering an ants’ nest (Erber 1988, and references therein). Within clytrines, adults of *Hockingia* exhibit an extraordinary morphology indicative of a strong integration with ants; yet, when considering the larvae or pupae, they seem to exploit a protective strategy, solely relying on the protection offered by their cases and a plug-like head. Important evidence to solve this problem might come from the study of known or new myrmecophilous organs, present in camptosomate beetles that would provide appropriate compensation for the ants. Nevertheless, according to present evidence, it is possible to find a plethora of relationships, from mutualism to parasitism. Below we introduce some interesting aspects of the evolutionary challenge faced by cryptocephalines living with ants.

The reports to date suggest some degree of specialization in non-*Clytra* species. *Megalostomis dimidiata* is an *Atta* specialist; *Anomoea*, *Clytrasoma* (Fig. 1D), *Hockingia* (Fig. 1A), and *Isnus petasus* are all *Crematogaster* specialists; *Tituboea biguttata* are harvester ant (*Messor*) specialists; and *Smaragdina concolor* (Fabricius) are known from the pavement ant *Tetramorium*. With respect to these ant species, some aspects of their biology may help explain this degree of specificity. Both *Atta* and *Messor* have long-lived nests, in that they typically remain in one location for several years (Hölldobler and Wilson 1990), and this is largely afforded by having a nearly constant source of food (fungus in the case of *Atta*; seed caches in the case of *Messor*). This sort of stable environment could allow for long-term myrmecophilous relationships to evolve, and in fact, *Atta* species typically harbor a large number of commensal species living among their nests (Waller and Moser 1990). It is also quite intriguing considering that the root node of *Atta* has been estimated to be some 8 my old, more or less a newcomer in evolutionary time, which means its inquiline (*Megalostomis dimidiata*) is likely to be even younger than that.

Regarding the strength of currently known Cryptocephalines/ants associations, Selman (1988) sorted the life histories of the following assemblages: *Clytra*/*Formica*, and *Hockingia*, *Isnus*/*Crematogaster* as cases of commensalism, or perhaps parasitism. The author also remarked the importance of the stable temperature within the ant nests as a clear advantage for cryptocephalines. Hitherto, even if these are the most studied cases, we do not know much about the organisms.

Another unusual pattern to emerge from our synthesis is the case of *Dorylus*, a genus that includes army ants, which do not construct a typical ground nest like many other formicids, but instead, a bivouac as needed, and remain constantly in search of prey items (Hölldobler and Wilson 1990, and references therein). Jolivet (1952) offers a very intriguing observation of an unidentified Clytrini whose larvae migrates with a *Dorylus* species; how a relationship such as this can evolve is quite fascinating, but certainly requires further verification beyond this single reference. *Labidostomis taxicornis* is the only known cryptocephaline to be associated with a Dolichoderinae (*Tapinoma erraticum*). Dolichoderines are some of the most notoriously invasive species of ants (entering non-native habitats/regions), with species such as *Tapinoma sessile* and *Tapinoma melanocephalum* essentially found worldwide. Considering the commonality of *Tapinoma* species, it is reasonable to expect that we would have recorded more cryptocephalines from this group of ants by now if the species actually existed. This dearth of cryptocephaline species associated with such a common, widespread group suggests the relationship between *Labidostomis taxicornis* and *Tapinoma erraticum* is something unique. This may also apply to *Smaragdina concolor*, which has been associated with the highly invasive pavement ant (*Tetramorium caespitum*). In this case, it would be fascinating to conduct additional fieldwork to determine if the beetle has been able to track its host ant into novel geographic regions, or if it is restrained to the aboriginal home of the ant.

Summarizing, existing evidence indicates multiple routes to myrmecophily in cryptocephalines, even if adults are above-ground herbivores and occasionally interact with ants; their immature stages (eggs, pupae and larvae) are the most exposed stages in terms of ant interaction. Myrmecophilous cryptocephalines can be found in subterranean (e.g. *Megalostomis* larvae found at 2.5m inside *Atta* nest), arboreal (e.g. *Isnus* in *Acacia* ant nests), and terrestrial (e.g. *Pachybrachis* on external debris of *Atta* nests) habitats.

**Figure 3.**
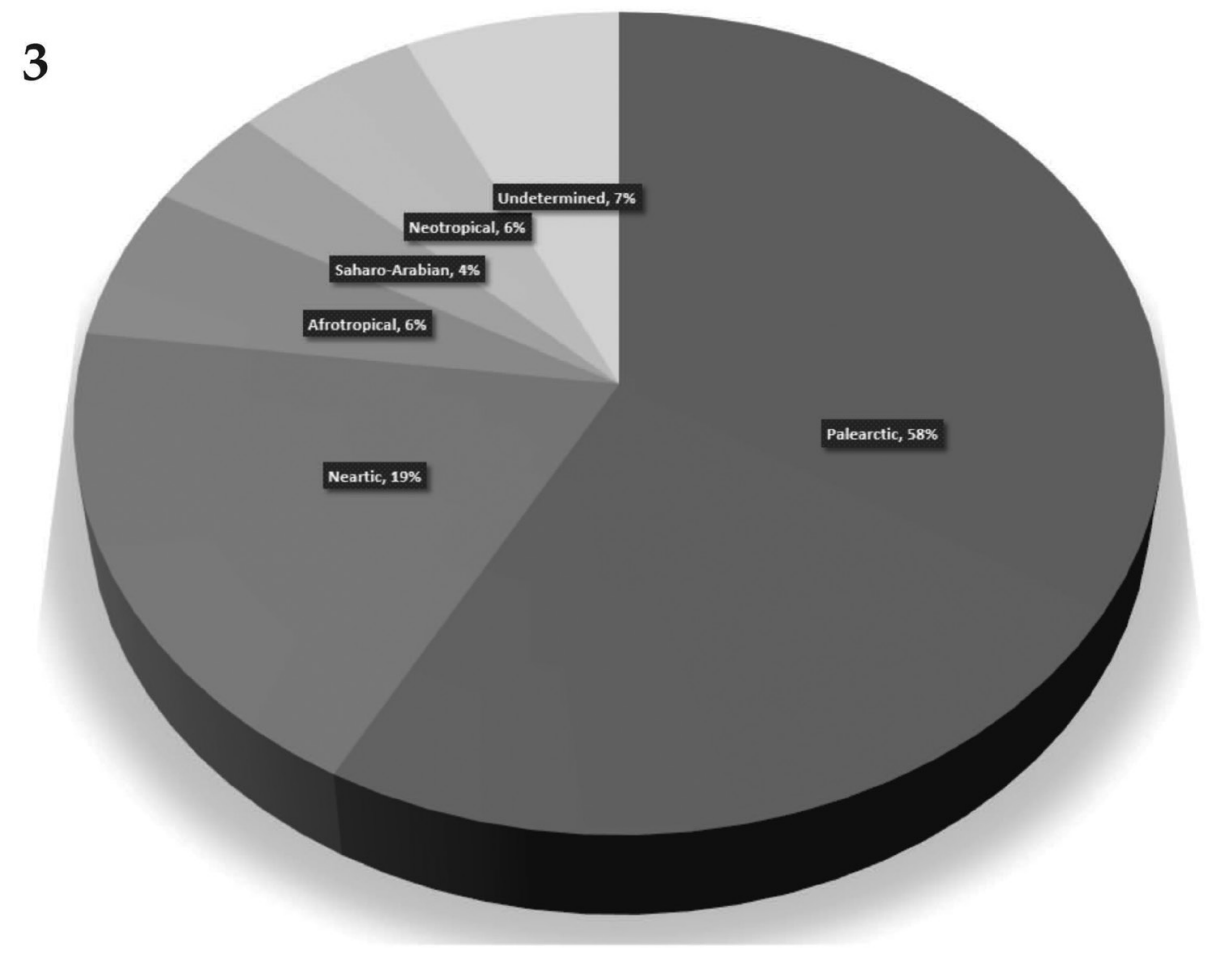
Ant association records by region. The percentage values indicates the fraction of ant association records known for each zoogeographic region.

*Geography of ant association* (Table 3). While the origin of myrmecophily is thought to be monophyletic among Old World genera of Clytrina (Schöller and Witte 2007), it is currently unknown if myrmecophily was inherited or newly acquired by New World cryptocephalines. To date, only two genera of Cryptocephalini have been cited as myrmecophilous in the New World. So far, no records of beetle/ant association have been found for Australian, Madagascan, Oceanian, Oriental, Papua-Melanesian, and Sino-Japanese regions (*sensu* Holt et al. 2013) (Fig. 3). The most remarkable specialized ant interactions known today (i.e. involving complex myrmecophilous organs and myrmecomorphy) are known for the Afrotropical region: Clytrini (*Hockingia*) and Cryptocephalini (*Isnus*), both genera related to *Crematogaster* ants. It is interesting to remark that *Crematogaster* is almost globally distributed today, and have apparently achieved this large distribution range by successive dispersion events since their origin in the mid-Eocene (Blaimer 2012). Actually, in most cases, the ants that have been associated with cryptocephalines are widely distributed, which suggests further records of myrmecophily are to be found for the also widely distributed cryptocephalines. The latter being especially true for the Neotropical region, which is mostly unexplored for myrmecophilous cryptocephalines, despite of its richness in endemic species and genera of ants (Holdobler and Wilson 1990), particularly in the tribe Attini. Finally, some other interesting patterns have been revealed in this study that require further investigation: although there is no myrmecophilus cryptocephaline recorded in Australia, there are three ant genera there that do host cryptocephalines in other parts of the world; even though the species of the dolichoderine *Tapinoma* are cosmopolitan, there is only one extant record for the genus.

**Table 3.** Genera of Cryptocephalinae by region and ant subfamily. Note all genera belong to the tribe Clytrini except for those marked with (*), which belongs to the Cryptocephalini.

Region	Formicinae	Myrmicinae	Dolichoderinae	Dorylinae	Undet.
Afrotropical	*Hockingia*	*Hockingia*, Isnus*, + undet.	-	Undet.	-
Nearctic	*Anomoea*, *Coscinoptera*	*Anomoea*, *Megalostomis*	-	-	*Anomoea*, *Coscinoptera*, *Lachnaia*, *Megalostomis*, *Saxinis*
Neotropical	*Megalostomis*, Pachybrachis*	*Megalostomis*, Griburius**	-	-	*Helioscopa*
Oriental	*Clytrasoma*				
Palearctic	*Clytra*, Cryptocephalus*, *Lachnaia*, Pachybrachis*, *Tituboea*, + undet.	*Clytra*, *Smaragdina*, *Tituboea*	-	-	*Clytra*, *Macrolenes* Cryptocephalus*, *Lachnaia* Pachybrachis*
Saharo- Arabian	*Clytra*	*Lachnaia*, *Tituboea*	*Labidostomis*	-	-

### Calibrating the evolutionary history of ants and Camptosomata

Wilson and Hölldobler (2005) pointed out three major events in the evolution of ants: 1) early radiation during mid-Cretaceous aided by complex ground litter (angiosperms assembled); 2) a dominance of ants in the angiosperm-rich tropical forests during the Paleogene; and 3) diet shift events in the dolichoderines, formicines, and certain myrmicines. These events triggered expansion from tropical forest and general predation, into the canopy and into xeric environments and more specialized feeding behaviors.

According to Brady et al. (2006) myrmecines and formicines are sister-groups, and they split around 75–84 mya (but see Moreau and Bell 2013), and some of them exploit symbiotic relationships. This evolutionary picture favors the scenario for the acquisition of myrmecophily in casebearer species and a date of origin in the upper Cretaceous. The older fossil evidence of cryptocephaline larvae (in their cases) include specimens in Dominican Miocene (20 mya) and Baltic (45 mya) ambers (Grimaldi and Engel 2005, Chaboo et al. 2009, Bukejs and Chamorro 2015), thus the minimum age of case-bearing behavior dates to 45 mya. This data is compatible with Wang et al. (2014), yet it differs from other studies on leaf beetles phylogeny (i.e. Hunt et al. 2007, Gómez-Zurita et al. 2007), which date the origin of Chrysomelidae and Cryptocephalines much later.

Table 2 clearly shows that nearly all of the known records of myrmecophily in the cryptocephalines belongs to the Myrmicinae + Formicinae clade, whose common ancestor date from Mid-Cretaceous, 125–75 mya (Brady et al. 2006, Moreau et al. 2006, Geiselhard et al. 2007). The Dolichoderinae clade has an estimated origin of 65 mya (Ward et al. 2010), and the tribe Tapinomini seems to have originated in the Paleotropics. Ergo, host ants might have been available early during the evolution of Cryptocephalinae, a lineage that began its diversification about 93 mya (Wang et al. 2014).

The evolutionary history of formicoid ants date back to the Upper Cretaceous period (Brady et al. 2006, Moreau and Bell 2013), with genus-level divergences being established later in the Eocene, 50–35 mya; this is also supported by their common presence in amber fossils (Grimaldi and Agosti 2000, Moreau et al. 2006, Dunn et al. 2007, Grimaldi and Engel 2005, LaPolla et al. 2013). Furthermore, the origins of myrmecochory (seed dispersal by ants) were also being established at this same time period (Dunn et al. 2007), which include the associations with Formicinae and Dolichoderinae, and the so called tree-loving ants (*Crematogaster*) which are well known canopy-dwelling herbivores (Rico-Gray and Oliveira 2007). Leaf-cutting Attini are far more recent, about 7–16 mya (Schultz and Brady 2008). Attines are limited to the New World, and most of the 12 genera and 190 species occur in the tropical portions of Mexico, Central and South America (Hölldobler and Wilson 1990). These ants form large, mature colonies with great diversity of myrmecophilous species. Certain leaf-cutting ants, such as species of *Atta*, house a large number of “nest guests”, for which interactions with the host (ants) are poorly understood (Waller and Moser 1990). The sophisticated habit of culturing and eating fungi by these fungus-growing ant species has evolved in other insects groups as Macrotermitine (termites) and certain wood-boring beetles. So far no case of termitophily has been reported for Cryptocephalines; further, termitophily has been regarded as extremely rare and accidental for Chrysomelidae in general (Jolivet and Petitpierre 1981). According to Korb (2008), social evolution was independently acquired by ants and termites lineages, the author remark that one of the major differences is that termites do not depend on food provisioning activities for their hemimetabolous brood; contrariwise ants has become excellent food providers in order to feed their brood. The latter might be thought as a key innovation in ant evolution, which might have driven the origin of myrmecophily within Cryptocephalines searching for food and shelter in ant nest. In this regard, we hypothesize that the nature of food items available inside the termite nest, as well as the effectiveness of the termite defense mechanisms (Puker et al. 2015, and references therein) might explain the absence of termitophily in Chrysomelidae, a relationship which has in fact been exploited by other lineages of Coleoptera as Elateridae, Passalidae, Melyridae, Scarabaeidae, Tenebrionidae, and Carabidae (Costa and Vanin 2010).

A parallel can be traced between the evolution of ants and cryptocephalines regarding climatic preferences. As mentioned above, the last major evolutionary event in ant evolution, according to Wilson and Hölldobler (2005), was the invasion into the canopy niche, aided by diet shift that allowed certain ant groups to colonizing xeric environments. A similar process can be inferred for the diet shift already discussed above in myrmecophilous casebearers: although extant evidence indicates that these might have occurred simultaneously, the precise interconnection of these processes need to be further analyzed to conclude what sort of evolutionary process predominates in such system. Taking Clytrini as an example, most species are characterized as inhabiting temperate, xeric regions (Lopatin 1999). Furthermore, Agrain and Roig-Juñent (2011), based on new and previous evidence, hypothesized a tropical/subtropical origin for this tribe. According to Cloudsly-Thompson (2001), the Tenebrionidae and, to a lesser extent, the Chrysomelidae, are among the most successful animals of xeric environments, and species of these groups are often the only ones to be seen at-large during the day. Jolivet (2004) analyzed adaptations of Chrysomelidae to xeric regions and he mentioned some potential adaptations of clytrines, such as omnivorous larvae, polyphagous adults, lack of potential competitors (i.e. the author mentions that less than 2% of Chrysomelids live in arid zones), and finally, ant-host protection in subterranean nests (microclimatic, hygienic, and enemy-free space). Schöller (1995) also proposed, as an adaptation to xeric environments, the use of the rectal sclerites as water retention organs. This is a morphological trait exhibited only by the females, and not being shared by males. However, males are short-lived compared to females in many insect species, consequently male longevity may not be adaptive. Moreover, the oviposition process may require a more sophisticated water retention mechanism for the females. We have also observed that most species within the subtribe Megalostomina have thicker elytra (relative to other clytrines in the Neotropical region), such as *Anomoea* or *Lachnaia*; this might help in water retention, in a similar way as the subelytral cavity in Tenebrionidae and some other beetles adapted to arid environments (Cloudsly-Thompson 2001 and references therein). Also, Monrós (1953) mentions that adults of *Megalostomis gazella* Lacordaire rest for periods during daytime, and this, together with the subterranean habits of the larvae, might be an adaptive means for living in climatic extremes.

*Key evolutionary steps in Camptosomata.* Case-bearing and its correlated behavioral and morphological characters are a complex synapomorphy distinguishing the clade Cryptocephalinae + Lamprosomatinae within Chrysomelidae. It is the most obvious defense mechanism of these immature stages (Poinar 1996) and represents a fundamental evolutionary step within leaf beetles. The habitat shift from living in plants to living inside arboreal or subterranean ant nests is an extraordinarily complex problem. A comprehensive phylogenetic analysis of Camptosomata currently underway by a team of researchers, including a number of authors of this paper, hopes to shed some light on the evolution of the group.

Recently, Parker and Grimaldi (2014) described the earliest known specialized myrmecophile, a rove beetle (Staphylinidae) that dates to the early Eocene (52 mya). This indicates that myrmecophily is an ancient evolutionary phenomenon. The detailed study of fossil larval morphology in Cryptocephalines might also provide evidence of specialized morphology indicative of myrmecophily behavior and it would establish a minimum age for myrmecophily within cryptocephalines. Yet, even though the immature stages of cryptocephalines have been studied (Chamorro 2014b, and references therein) and are well preserved in amber fossils, no data on possible myrmecophily specialized structures or chemical substances are currently known.

## Conclusions

Unraveling the evolutionary patterns of the habitat and diets shift in adults and larvae, as well as understanding, the multiple behavioral and morphological adaptations of ant-loving cryptocephalines will require extensive field work and inter-disciplinary approaches. The relationship with ants suggests the acquisition of ethological and morphological characters that are currently poorly studied. Some basic research activities include: field observations; experiments using artificial ant nests; and detailed morphological studies of the adults and immature stages. Also, the study of the degree of the association (facultative vs. obligate), the effects of this association on host plant choice (i.e. tropic selection mediated by ants), and the possibility of linked cladogenesis between ants and cryptocephaline phylogeny and diversification. Cost-benefit analysis will evaluate the role of each member in an association. Description of life cycles, as well as detailed anatomical studies of all stages are necessary, especially the study of myrmecophilous organs and the possible chemical cues involved. The behavioral, morphological, and chemical adaptations of cryptocephaline myrmecophiles are promising areas for further research.
